# Head Anticipation During Locomotion With Auditory Instruction in the Presence and Absence of Visual Input

**DOI:** 10.3389/fnhum.2019.00293

**Published:** 2019-08-28

**Authors:** Felix Dollack, Monica Perusquía-Hernández, Hideki Kadone, Kenji Suzuki

**Affiliations:** ^1^School of Integrative and Global Majors, University of Tsukuba, Tsukuba, Japan; ^2^Artificial Intelligence Laboratory, University of Tsukuba, Tsukuba, Japan; ^3^NTT Communication Science Laboratories, Atsugi, Japan; ^4^Center for Innovative Medicine and Engineering, University of Tsukuba Hospital, Tsukuba, Japan; ^5^Center for Cybernics Research, University of Tsukuba, Tsukuba, Japan; ^6^Faculty of Engineering, Information and Systems, University of Tsukuba, Tsukuba, Japan

**Keywords:** head anticipation, locomotion, eyes closed, auditory perception, motor planning

## Abstract

Head direction has been identified to anticipate trajectory direction during human locomotion. Head anticipation has also been shown to persist in darkness. Arguably, the purpose for this anticipatory behavior is related to motor control and trajectory planning, independently of the visual condition. This implies that anticipation remains in the absence of visual input. However, experiments so far have only explored this phenomenon with visual instructions which intrinsically primes a visual representation to follow. The primary objective of this study is to describe head anticipation in auditory instructed locomotion, in the presence and absence of visual input. Auditory instructed locomotion trajectories were performed in two visual conditions: eyes open and eyes closed. First, 10 sighted participants localized static sound sources to ensure they could understand the sound cues provided. Afterwards, they listened to a moving sound source while actively following it. Later, participants were asked to reproduce the trajectory of the moving sound source without sound. Anticipatory head behavior was observed during trajectory reproduction in both eyes open and closed conditions. The results suggest that head anticipation is related to motor anticipation rather than mental simulation of the trajectory.

## 1. Introduction

Perhaps one of the most distinct human characteristics is the ability to move on two feet. Even though it is a highly automated task (Doyon et al., 2009), human locomotion is a complex phenomenon that requires multisensory information. To achieve adaptable posture-gait control, somatosensory, visual and vestibular sensations are integrated (Takakusaki, 2017). However, the exact mechanisms that make it possible are still under debate. Therefore, it has been a challenge to replicate bipedal walking in artificial agents (Westervelt et al., 2007).

One of the mechanisms thought to govern steering in human gait is head anticipation. Head anticipation is the change of the head toward the future heading direction. Anticipation has first been connected to the steering of locomotion (Grasso et al., 1996). Five participants were instructed to walk along circular trajectories of different diameters, both at light and while blindfolded. The results indicated that steering of locomotion along a pre-planned trajectory is generated and controlled by an anticipatory guidance of the direction of the head. In a later experiment, six participants were asked to walk forward, and backward around an obstacle, both with eyes open and with eyes closed. The results showed head anticipation in both visual conditions and walking directions (Grasso et al., 1998). Walking of participants along complex trajectories such as an eight and limacon shape in light and complete darkness with eyes open also showed head anticipation (Authie et al., 2015). Moreover, the spatial and temporal relationship among the direction of gaze and body segments have been explored while walking along complex trajectories such as a clover leaf shape, an eight shape and a limacon. The anticipatory guidance sequence starts with the eyes glancing at the new direction before head, shoulders, torso and pelvis rotate with small delays toward the future heading direction (Kadone et al., 2010; Bernardin et al., 2012). A similar order of changes in head and torso orientation that was unaffected by absence of vision has been reported in an experiment to evaluate body trajectory and segment orientation along curved trajectories (Courtine and Schieppati, 2003). Although it is clear that head anticipation occurs, the purpose of this anticipatory behavior is still under debate.

As summarized by Authie et al. (2015), the possible functions of this phenomenon include motor anticipation, a mental simulation of the trajectory, and an active vestibular and proprioceptive process contributing to spatial perception during self-motion. Among these options, vision provides the central nervous system with direct cues from participants' extra-personal space and appears to be the most important sensory modality during curved walking (Courtine and Schieppati, 2003). However, the results under different visual conditions indicate that the fundamental function of head anticipation is independent of vision (Takei et al., 1997; Grasso et al., 1998; Courtine and Schieppati, 2003; Authie et al., 2015). If this anticipation is in fact independent of the visual input, head anticipation should also be observable without relying on visual representations, including the instructions provided on the trajectories to follow. If head anticipation does not merely assist the brain in exploiting visual information, but rather is part of motor anticipation, this phenomenon should be observable even when visual representations are not used to plan the trajectory to follow. Therefore, it is important to investigate the influence of the absence of vision on head anticipation.

Spatial cognition and navigation, similar to head anticipation, are often studied using the visual sensory modality only. However, there are a few exceptions where visual input has been substituted with auditory input. Gori et al. (2017) investigated the auditory perception and navigation skills in 10 blind and 10 sighted individuals. Their paradigm required the participants to reproduce a trajectory after memorizing it by receiving only auditory cues from a moving sound source while they remained stationary. Their results indicate that blind individuals have trouble detecting shapes due to a compromised Euclidean representation of space. Furthermore, blind participants compress shapes and tend to reproduce circular trajectories. In another study, spatial memory in humans was investigated with an acoustic version of the Morris water-maze (Viaud-Delmon and Warusfel, 2014). Similarly to the hidden platform below the water surface in the original water-maze task (Morris, 1981), eleven blindfolded participants had to find a hidden sound target. The sound target became audible only when they were inside a certain perimeter. A set of spatially fixed sound sources acted as auditory landmarks to help participants orientate in space. The results revealed that auditory and motor cues constitute relevant enough information to create a spatial representation without visual information. Moreover, Karim et al. (2017) hypothesized that auditory inputs are used as environmental spatial landmarks during locomotion. Dynamic balance of eight blindfolded participants was assessed using the Fukuda-Unterberger stepping test in three auditory conditions: silence, white noise played through headphones, and white noise played through a loudspeaker. Their results suggest that presence of sound dramatically improves the ability to ambulate when vision is limited, as long as sound sources are located in the external environment. Yelnik et al. (2015) were looking to increase the difficulty in the Romberg test under different visual conditions. The Romberg test is a standard examination of neurological proprioceptive disorders in which examinees' body sway is measured standing erect with feet together with open and closed eyes. Patients with proprioceptive disorders will sway more and lose balance with eyes closed. They tested 50 participants in two protocols with eyes open, eyes open wearing black goggles, eyes open wearing white goggles, and eyes closed. Their results indicate that walking without any visual input with closed eyes is easier than walking with eyes open in darkness. They argued that visual dependence has been demonstrated among children and adults when confronting a new motor difficulty. Furthermore, closing their eyes might give the participants access to mental imagery of well known space, rather than relying on perturbed visual input.

In this line of thought, we proposed to investigate locomotion with auditory instead of visual instructions and in two visual conditions, eyes open and eyes closed. We hypothesize that (1) healthy sighted participants can memorize and reproduce auditory-guided locomotion trajectories regardless of any visual input. This includes both instruction of the shape and visual input during sound following and reproduction. Furthermore, we hypothesize that (2) head anticipation can be observed even in absence of these visual cues.

First, we validate our setup by evaluating the ability of participants to locate virtual sound sources. We asked participants to localize spatially static sound sources with headphones and walk to their origin (Loomis et al., 1990). Second, we adopted the paradigm from Gori et al. (2017) to investigate head anticipation during auditory instructed locomotion, as it does not rely on visual information. To train the expected trajectory, we presented participants with a moving virtual sound source. The sound was presented with wireless headphones and real-time spatial audio reproduction software to avoid possible variations in the instruction (Gori et al., 2017) and to increase reproducibility. Instead of listening passively, we asked participants to actively follow the trajectory of the source, because it was suggested that an auditory spatial representation can be formed based on sensory-motor and auditory cues alone (Viaud-Delmon and Warusfel, 2014). This training was repeated twice for every trajectory. Third, we asked participants to reproduce the trajectory of a moving sound source. During the reproduction-task the sound was removed, to avoid systematic eye movement induced by sound (Braga et al., 2016).

The results show that our setup produces spatial virtual sounds that can be localized with an accuracy of ~10 centimeters. We further could observe that participants were able to closely follow moving sound sources. Participants were able to reproduce auditory instructed trajectories in the presence of vision. However, in the absence of vision participants were only able to reproduce circles and not the more complex eight shape. Finally, we could show the existence of head anticipation in presence and absence of vision during auditory instructed locomotion.

## 2. Materials and Methods

### 2.1. Participants

Ten volunteers (5 female, mean age 27.1 years old, *SD* = 3.5) took part in the experiment. The study was carried out at the university hospital of the University of Tsukuba. All participants gave written informed consent in accordance with the Declaration of Helsinki. The protocol was approved by the ethical committee of the University of Tsukuba (2018R259).

### 2.2. Apparatus

The experiment took place in a room with dimensions 15 by 5 m. A Vicon motion tracking system (VICON MX, Vicon, UK) was used to record head and torso position and movements. Participants were wearing three reflective motion tracking markers on their shoulders (right and left acromion) and the back (cervical vertebrae 7).

Furthermore, they wore a radio frequency (RF) wireless headphone (UHF wireless headphone, Ansee, China) to receive the sound stimulus. This headset was equipped with four reflective motion tracking markers to record the head position and orientation and forward it to a MacBook Pro (2.7 GHz Intel Core i7, 16 GB RAM, Mid 2012, OS X El Capitan, Apple, US) running an open sound control (OSC) server written in Python. This server controlled the auralization with an instance of soundscape renderer (Geier et al., 2008). The auralization used head-related transfer functions (HRTFs) that were part of soundscape renderer. The HRTFs were recorded in an anechoic chamber from the torso and head simulator FABIAN (Lindau and Weinzierl, 2006) and equalized for a typical pair of headphones. The base station of the RF headphones was connected to the headphone jack of the MacBook.

Additionally, eye movement was recorded using electro-oculography (EOG). Participants wore disposable electrodes (Kendall ARBO H124SG, CardinalHealth, US) above and below their right eye (vertical direction) and outside their right and left eye (horizontal direction). A fifth electrode on the mastoid behind the right ear served as reference. The electrodes were connected to a Shimmer3 ECG/EMG Bluetooth device (Shimmer, Ireland) that was attached with velcro to the outside of the right cup on the headphones. The shimmer device was streaming data to a Dell Alienware laptop (2.8 GHz Intel Core i7, 16 GB RAM, Windows 10). In conditions with eyes open, participants also wore eye tracking glasses (Glasses 2 Pro, Tobii, Sweden). The glasses provided a WLAN access point to which the Dell Alienware laptop connected. An OSC server forwarded control and trigger commands to the glasses. The position of the stimulus and the recording of the participants' responses were controlled by custom-written C++ software on a second MacBook Pro (2.4 GHz Intel Core 2 Duo, 16 GB RAM, Mid 2010, macOS High Sierra). To avoid any kind of visual stimulation and thus remove all external light, participants were asked to close their eyes and wear a pair of swim goggles covered with multiple layers of black electrical insulation tape.

### 2.3. Stimuli

The basis for the virtual sound source was a sinusoid created with Matlab v2017b (Mathworks, US). The signal had a frequency of 250 Hz at a sampling rate of 44.1 kHz and had a duration of 5 min. The stimulus level was set to a comfortable sound level close to normal speech for a distance of 0 m. The auralization software adapted the stimulus level separately for left and right ear depending on the head orientation of the listener and the distance to the sound source.

### 2.4. Task

**Figure 1:**
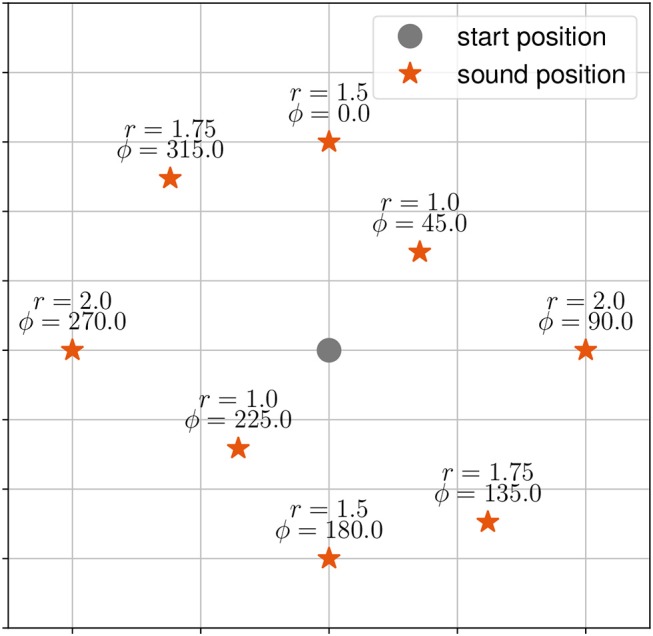
Target stimuli (red asterisk) and start position of the participant (grey dot) as used in the static sound task. Target stimuli are given with distance *r* and angle ϕ to the start position.

There were three tasks. During the first task, henceforth called the “localization-task,” participants were asked to localize and walk to the origin of a stationary virtual sound source. The target stimuli were presented at eight positions (distance in meter *r*, angle in degrees ϕ) starting at 0° with clockwise increments of 45° (see Figure 1).

**Figure 2:**
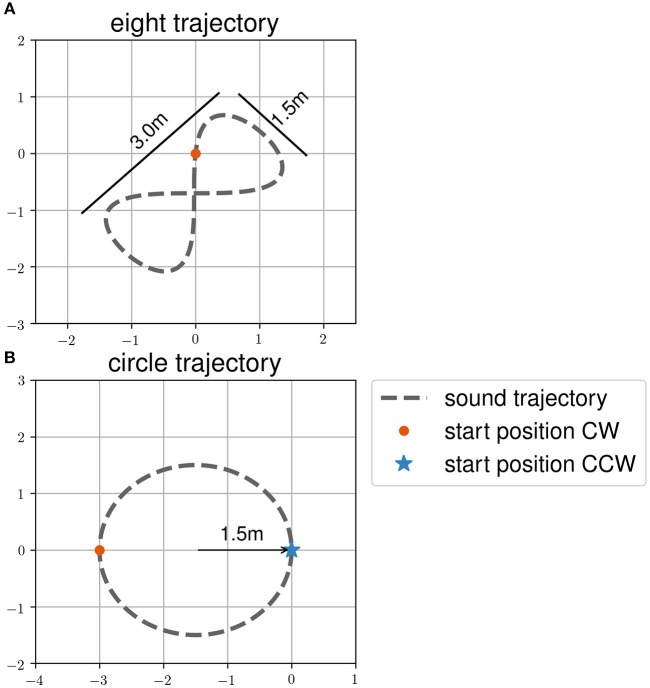
Target trajectories **(A)** eight and **(B)** circle and start positions of the participants as used in the dynamic sound task. Trajectories are shown as dashed line. Start positions of clockwise (CW) trajectories are shown with a red dot. Start positions of counter clockwise (CCW) trajectories are shown with a blue asterisk.

In task two, henceforth called the “following-task,” participants were asked to follow a moving sound source. They were also told that they would have to reproduce the trajectory without sound in a third task, henceforth called the “reproduction-task.” The trajectory order during the following-task was the same as during the reproduction-task. Participants had to follow the sound source on all trajectories twice before reproducing it once. The trajectories were (1) an eight, (2) a circle in clockwise direction, and (3) a circle in counter clockwise direction (see Figure 2). The order of the trajectories and sound targets during all tasks was randomized within two blocks of eyes open and closed trials. These blocks were counterbalanced among participants. Participants were in two groups, where one started with eyes closed, while the other groups started the experiment with eyes open. The room was lightened (environmental light during the day and neon light in the evening). The room was a therapy room used for gait rehabilitation with therapy tools visible.

### 2.5. Procedure

First, the participants were guided to the start position. Localization-task trials started with the playback of the sound. The trials ended with the participants signaling that they found the target position by saying “stop.” The following-task trials began with a virtual sound source playing in front of the participant. The sound then moved along a trajectory. After 60 s, the sound source stopped playing regardless of the participants' position. The participants repeated each following-task trial two times as preparation for the reproduction-task. In the reproduction-task, participants were instructed to start walking when the experimenter said “go.” The reproduction-task stopped whenever the participants signaled that they were satisfied with the reproduced trajectory. When there was risk of collision with a wall, the experimenter stopped the participants. This happened in seven trials with eyes closed out of a total of 60 reproduction trials. In those cases, the participants were asked to repeat the reproduction. The localization-task lasted roughly 25 min while the following-task and reproduction-task together took around 20 min per visual condition.

### 2.6. Data Analysis

The data analysis and visualization was performed with Python (version 3.6). R (version 3.4.3) was used for statistical analysis. EOG recordings were linearly detrended and low-pass filtered with a Butterworth filter at a frequency of 10 Hz. Eye tracker data was low-pass filtered with a Butterworth filter at 30 Hz. Recordings from the Tobii eye tracker were used to validate the correlation with the EOG signal. To estimate the gaze directions left and right only the horizontal EOG signal was used, since the eye tracker only works with eyes open. The detrended EOG signals were normalized to ±0.5. Then, these values were divided into three gaze direction areas to account for forward gaze. Eye movement to the right side shifted the negatively charged back of the eye toward the cathode of the EOG setup. This resulted in a negative signal for looking to the right and a positive signal for looking to the left. The forward gaze area thresholds were selected to cover 30% of the amplitude or ±0.15.

VICON data was used to calculate the position of the participant and to determine horizontal angles for head and torso. For the calculations, marker pairs were selected on the left and right shoulder and on the headphone. The position was calculated as the mean between the marker pairs. Orientation angles were calculated as the arctangent of the difference between marker positions in the x and y axes and smoothed by a first order Savitzky-Golay filter with a window length of 151 samples.

In the localization-task, the Euclidean distance between the end-point reached by the participants and the target position was calculated to access the localization error of the participants. The end-point of the trial was calculated as the average of the last 60 samples, equivalent to 1 s, before the participants signaled to stop playback. The localization error as well as the duration of the task were used to analyze for possible effects of sound source position and visual condition.

In the following-task, motion trajectories of the participants were linearly interpolated to the shortest valid trial. The error between the position of the participants and the sound source was then calculated as the average over all Euclidean distances of the interpolated signals from trials with sound. The effect of eyes open and closed was statistically analyzed using general linear mixed models and the R packages lmer and psycho (Makowski, 2018). Moreover, head movement has been analyzed for targeting the sound source position.

In the reproduction-task, the delay between horizontal orientation angles of head and torso was calculated by means of cross-correlation to evaluate the existence of anticipatory head behavior. Head anticipation has been statistically analyzed by running Wilcoxon rank tests with the delays between head and torso orientation.

## 3. Results

**Figure 3:**
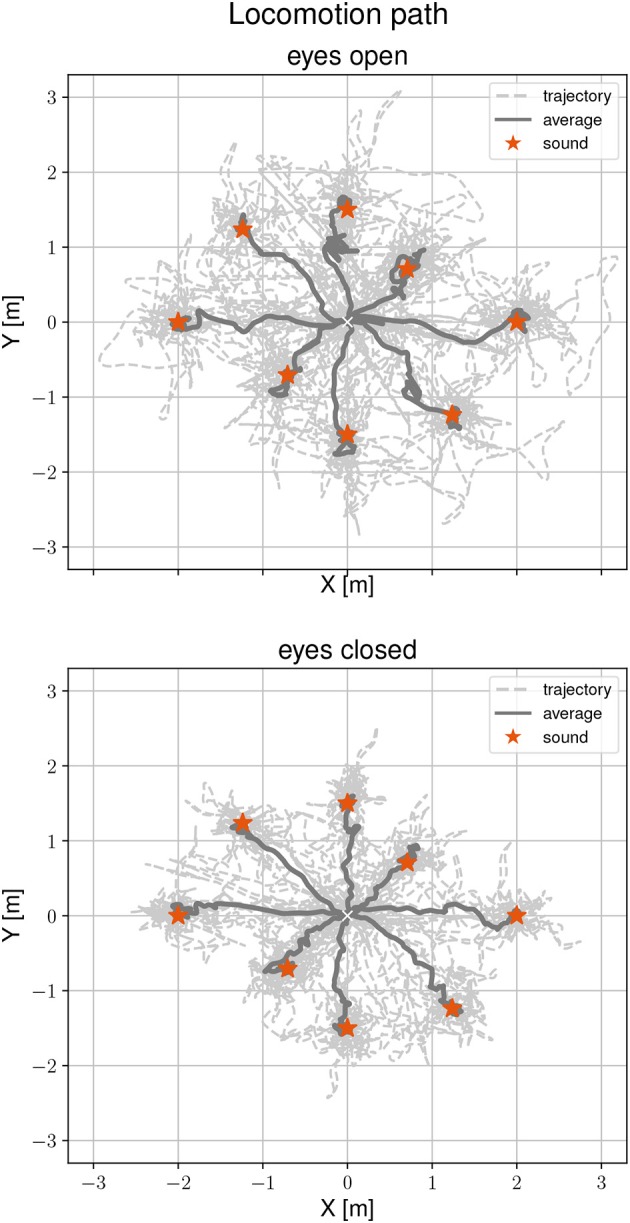
Locomotion trajectories during the static sound localization task with eyes open **(Top)** and eyes closed **(Bottom)**. The participants' trajectory is represented with a dashed line, the average path with a solid line, and the sound position with a red asterisk.

Figure 3 shows locomotion trajectories of all participants during the localization task for both visual conditions. Sound positions are marked by a red asterisk. The average trajectory from the start position to the sound source origins is drawn as a solid line. A general mixed linear model was fitted to the localization error as a dependent variable to asses differences between visual conditions, target positions and the interaction between them. The effect of eyes closed is small (β = -0.03, *SE* = 0.04, 95% CI [−0.1, 0.05], η^2^ = 0.12) and can be considered as significant [*F*_(1, 72)_ = 9.73, *p* < 0.01]. The grand mean for accurate determination of the sound source position was 0.14 m. By visual condition, the means were 0.16 m (range 0.02–0.48 m) with eyes open and 0.12 m (range 0.01 and 0.46 m) with eyes closed. Performing the task with eyes closed is more accurate by one-third (32%) compared to eyes open. There were no significant effects of target position on localization error [*F*_(7, 63)_ = 0.37, *p* = 0.92], as well as no interactions between visual conditions, target position and localization error [*F*_(7, 72)_ = 0.83, *p* = 0.57]. To find the static sound sources, participants needed between 11.56 s and 62.85 s with eyes open and 11.32 s and 79.91 s with eyes closed. There were no significant effects between visual condition and task duration [*F*_(1, 135)_ = 1.43, *p* = 0.23, η^2^ = 0.001] or between target position and task duration [*F*_(7, 135)_ = 0.79, *p* = 0.6]. There was a significant interaction between visual condition, target position and task duration [*F*_(7, 135)_ = 2.33, *p* < 0.05]. However, this interaction was only found in the 225° direction.

**Figure 4:**
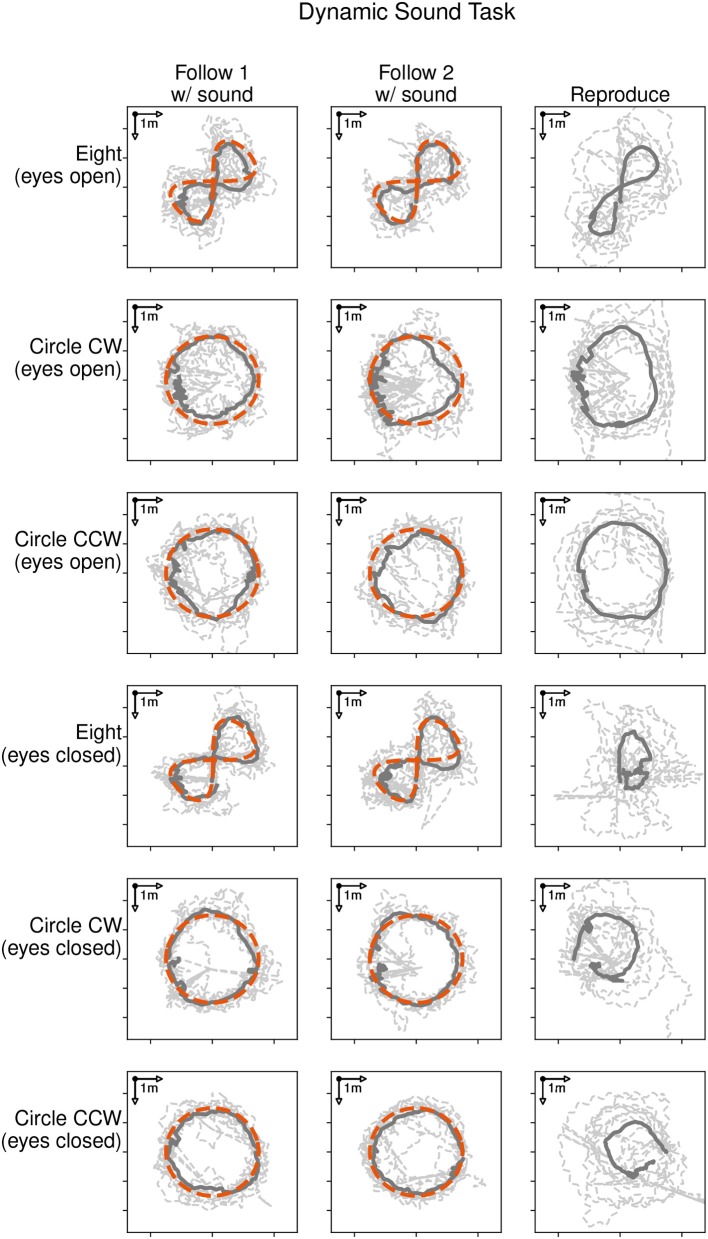
Locomotion trajectories of all participants with eyes open (rows 1 to 3) and eyes closed (rows 4 to 6) during the following-task (left and middle column) and the reproduction-task (right column). The participants' trajectory is represented with a dashed line, the average path with a solid dark line, and the sound source trajectory as red dashed line.

Figure 4 shows the locomotion trajectories of all participants during the following-task (left and middle column) and reproduction-task (right column). Each trajectory is represented with a dashed line. The average trajectory followed and reproduced by all participants is shown as a solid dark line. Each target trajectory type is represented with a row for the eyes open condition (rows 1 to 3) and the eyes closed condition (rows 4–6). The target trajectory (red dashed line) was presented with a moving sound source and followed by the participants during the following-task. There was no sound source in the reproduction-task, as participants were asked to reproduce the previously shown trajectory. Thus, there is no depiction of the target trajectory in the graphic.

Participants were able to follow the moving sound source for all given trajectories. The average distances during the following-task were 0.60, 0.57, and 0.57 m in the eyes open condition and 0.58, 0.48, and 0.49 m in the eyes closed condition for the eight, clockwise and counter-clockwise circle trajectories respectively. The average durations taken to reproduce the three trajectories were 36.1, 37.4, and 34.2 s in the eyes open condition and 49.2, 47.2, and 51.8 s in the eyes closed condition.

**Figure 5:**
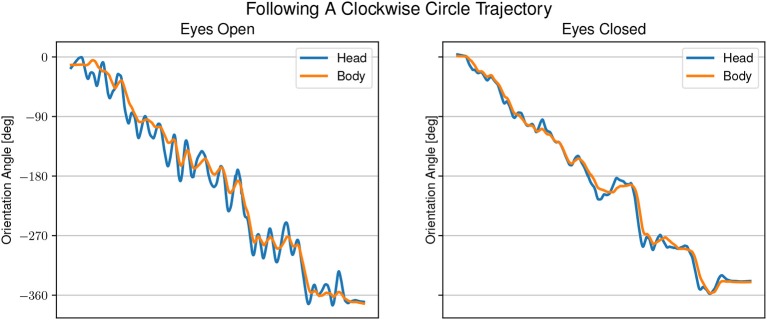
Example of head and body orientation angles of a single representative participant in the “following-task.” On the left in the eyes open condition, on the right in the eyes closed condition. A sinusoidal shift in head orientation can be seen compared to the body orientation.

**Figure 6:**
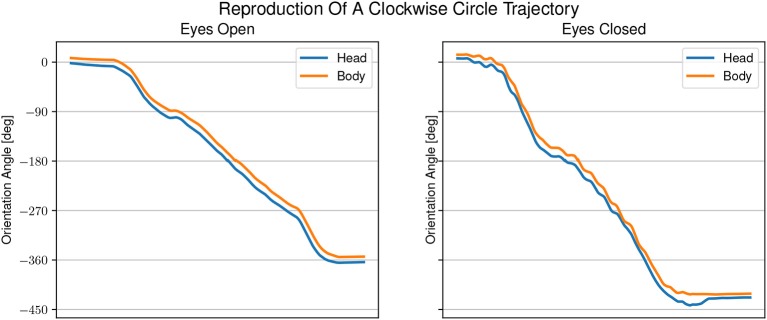
Example of head and body orientation angles of a single representative participant in the “reproduction-task.” On the left in the eyes open condition, on the right in the eyes closed condition. A constant shift to smaller angles can be seen for the head orientation compared with the body orientation. The shift in head orientation is larger with eyes open than with eyes closed.

Figures 5, 6 show examples of head and body orientation angles of a single representative participant in both visual conditions during the following-task and the reproduction-task respectively. For the data of the following-task, a general mixed linear model fitted to the error distance as dependent variable was used to asses differences between visual conditions, target trajectory, and the interaction between them. The effect of eyes closed was significant (β = −0.09, *SE* = 0.06, 95% CI [–0.21, 0.04], η^2^ = 0.65) and can be considered as medium [*F*_(1, 54)_ = 5.77, *p* < 0.05]. There were no significant effects between target trajectory and error distance [*F*_(5, 45)_ = 0.81, *p* = 0.55] and no interactions between visual condition, target trajectory and error distance [*F*_(5, 54)_ = 0.76, *p* = 0.58].

In the eyes open condition, changes in body angle are led by changes in head orientation with large overshoots of the head of up to 45°. The gaze direction appears to be aligned with the sound angle during phases when the head is moving away from the sound source. In the eyes closed condition, the head angle is changing closely together with, or slightly after a change in body angle. The difference between head and body angle with eyes closed is much smaller than in the eyes open condition, but still changing at the same time. The gaze is directed mostly toward the sound source.

**Figure 7:**
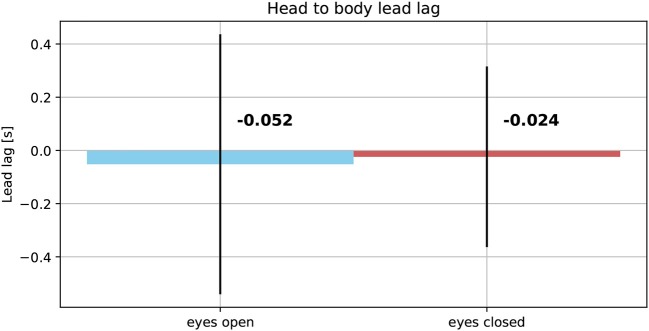
Average lead lag of head orientation angles over body orientation angles in eyes open **(Left)** and eyes closed condition **(Right)**.

The averaged reproduced shapes in the eyes open condition are clearly recognizable. In the eyes closed condition all averaged reproduced shapes look like circles. With eyes open the head orientation angle is constantly smaller than the body orientation angle. In the eyes closed condition the head orientation angle is also smaller than the body orientation angle. The gaze is constantly directed toward the inside of the trajectory. See Figure 7 for the average lead lag of head angle over body angle. One-sample Wilcoxon rank tests with continuity correction yield significant differences for the delays between head and body for both visual conditions from a mean larger or equal to 0 (eyes open: *Z* = −4.92, *p* < 0.001, eyes closed: *Z* = −3.45, *p* < 0.001). A paired samples Wilcoxon rank test with continuity correction indicates that the delays between head and body orientation in visual condition with eyes closed (*Mdn* = −0.024) are not different (*Z* = −0.30, *p* = 0.38) from the visual condition with eyes open (*Mdn* = −0.052).

## 4. Discussion

In this work, we have investigated locomotion with auditory instead of visual instructions. Participants went through two visual conditions to assess if head anticipation is observable in auditory instructed locomotion. For the sound instruction, we presented a virtual sound rendered according to the position of the participant. First, we validated this setup with a sound localization task, which confirmed that participants were able to localize the implemented virtual sound sources. The results also showed a higher localization accuracy in the eyes closed condition, compared to the eyes open condition. The two main hypotheses of this study were that (1) healthy sighted participants can memorize and reproduce auditory-guided locomotion trajectories regardless of any visual input and that (2) head anticipation can be observed even in absence of these visual cues. The participants were able to memorize and reproduce sound sources with our setup during the eyes open condition, but they were not so accurate with their eyes closed. Moreover, we found evidence supporting our main hypothesis for head anticipation in auditory instructed locomotion with and without visual input. Finally, we observed that this head anticipation does not occur in the presence of a sound target.

In a similar virtual sound localization task, Loomis et al. (1990) reported error distances between participants and sound sources in the range of 0.21 meter (0.15 m to 0.36 m) and searching time to find virtual sound sources from 9.6 to 25.2 s. Our results show a wider range with localization errors larger and smaller than reported by Loomis et al. (1990). However, the average localization errors are in accordance with the ones reported in their study. Half of the trials in Loomis et al. (1990) were done by participants with previous experience in tracking or homing to homing to virtual sound sources, whereas participants in this study had only one trial per visual condition to get familiar with the setup. This might explain the slightly larger range of error distances we measured. The on average smaller distance errors could be credited to the more sophisticated auralization software. Position updates and therefore the sound intensity were adjusted at a speed 10 times higher than in their study. Also, instead of rough approximations for the acoustic influence of the head, measured head related transfer functions from an artificial head were used as part of the software soundscape renderer. The large increase in time needed to finish the task compared to Loomis et al. (1990) could have emerged from the different actual room sizes used in the experiments. Since participants have seen the room and were aware of the spatial conditions, the size of the room might have led participants to perform more subtle movement and avoid quick walking out of expectation for sound targets to be closer. Loomis et al. (1990) conducted their experiment inside a gymnasium on an area of 225 m^2^, whereas our study was performed in a medium sized rectangular room within an area of 19.6 m^2^.

The larger variability of results in the eyes open condition could be the result of a conflict between visual and auditory perception. Vision retains a dominance in spatial localization under conflict conditions even if the visual input is not reliable (Warren, 1979; Bolognini et al., 2005). While trying to merge information from the visual and auditory systems, the virtual and therefore invisible sound source can be heard but not seen. Closing the eyes might signal the brain that there is no visual information to merge and relies more on the auditory system, leading to better results with closed eyes. This seems to be in line with results from a study that showed that no visual input during walking is better than with visual deprivation (Yelnik et al., 2015).

Next, we hypothesized that healthy sighted participants can memorize and reproduce auditory guided locomotion trajectories regardless of the visual condition. The average trajectory during the following-task with closed eyes shows less variance to the target trajectory than with eyes open. With open eyes, conflicting visual input in the cluttered environment might have been distracting. Participants had no problems reproducing the trajectories with eyes open. However, contrary to previous results the average reproduced shapes with eyes closed look like circles and were more similar to results of congenital blind participants (Gori et al., 2017). The differences in our setup compared to previous works are (1) the absence of reverberation (Viaud-Delmon and Warusfel, 2014; Gori et al., 2017), (2) a dynamic vs. static position of the listener (Gori et al., 2017), (3) in the case of a dynamic listener, no static spatial sound landmarks available (Viaud-Delmon and Warusfel, 2014), and (4) the expected trajectories were unknown beforehand. During listening to the sound trajectory with a static listener position an egocentric frame of reference can be assumed (Wang and Spelke, 2000; Loomis et al., 2001). The perceived directional cues and the loudness of the sound source provide information about the sound source position with respect to the listener. The sound source can then be triangulated through the listener's static position, the perceived sound position and either one or a combination of reverberation from other objects in space, changes in auditory cues of the moving sound source at different time steps and head movement. This seems to be sufficient for sighted individuals to build a mental representation in case of a static listening position. Although reverberation can degrade the localization accuracy (Shinn-Cunningham, 2000a), it still can be helpful for building a mental representation, as it provides information about the spatial dimensions of a room and relative position information about objects in it (Shinn-Cunningham, 2000b). To build a mental representation during self-motion, an allocentric frame of reference and objects that can act as reference are necessary (Heft, 1996; Wang and Brockmole, 2003; Waller and Hodgson, 2006; Zaehle et al., 2007). The available information from proprioception and the directional cues and the loudness of the sound source were not enough to inform participants about their position and orientation in space. This could have hindered participants to create an allocentric representation of space in the context of a moving sound source. To improve the results, future work has to increase the amount of acoustic information available by at least adding static sound landmarks as in Viaud-Delmon and Warusfel (2014) and use a room acoustic model that adds reverberation.

Finally, we hypothesized that head anticipation can be observed in auditory instructed locomotion regardless of the visual condition. During the following-task, we observed increased oscillations of the head instead of path anticipation. If the observed oscillations were due to the typically during gait expected head stride-to-stride oscillation (Brodie et al., 2015) the low-pass filter should have removed those or at least dampened their magnitude. It is more likely that the head oscillations were due to the presence of the auditory stimulus. Head oscillations during localization are performed naturally to reduce ambiguity in perception of sound that falls on the cone of confusion (Toyoda et al., 2011). The larger oscillations with open eyes compared to closed eyes as seen in Figure 5 are likely to be of the same nature as the differences between the visual conditions in the localization-task. Visual dominance and the low reliability of vision, due to the absence of a visual target, stand in conflict with the auditory input. This suggests that the presence of a target makes it difficult to proof the existence of head anticipation. In support of our hypothesis, we observed a consistent change in head orientation toward the participants' future body heading direction in the reproduction-task. Nevertheless, the effect was larger in the eyes open condition than in the eyes closed condition. Furthermore, contrary to other studies (Grasso et al., 1996, 1998; Authie et al., 2015), we did not find significant differences between head and body orientation in both visual conditions.

In their paper, Authie et al. (2015) outlined three possible functions of gaze anticipation: (a) mental simulation of the trajectory, (b) an active process contributing to spatial perception during self motion and (c) motor anticipation. If head anticipation serves as a mental simulation or expectation of the trajectory, we would expect this simulation to rely on some kind of internal spatial representation. Given the nature of the instructions in previous studies, these representations would be mainly visual. On the other hand, we took special care to avoid triggering an a priori visual representation, assuming that an internal spatial representation could also exist in form of an auditory representation. Integration of auditory and visual happens in different places in the brain depending on the availability of certain visual or auditory cues like a mouth or speech-like signals (Vander Wyk et al., 2010). A more general place that possibly marks the end of the audio-visual integration process is in the posterior superior temporal gyrus, as this structure seems to be most active when generating a merged percept under conflicting auditory and visual cues that are not necessarily speech (Erickson et al., 2014). However, our participants were not able to reproduce the more complex eight shape in absence of vision. This might be because of the mental load required for the participants to create a mental representation of each trajectory; or they were simply not able to build an auditory spatial representation with the given auditory cues. This result weakens the theory of anticipation being a contributor to spatial perception during self motion. It also allows for the conclusion that anticipatory behavior is unrelated to internal representations and therefore most likely does not function as mental simulation of the trajectory. The fact that head anticipation appears regardless of a possible absent internal spatial representation supports the hypothesis that the function of head anticipation is closely related to motor anticipation (Authie et al., 2015).

Anticipatory postural adjustment are probably controlled by the supplementary motor area and the premotor area, with poroparietal cortex involvement for accurate gait control in unfamiliar environments. Moreover, posture-gait control is dependent on sensory afferents and is most likely occurring in the cerebellum. The fastigal nuclei probably receives a copy of the spinal cord in addition to peripheral sensory information via spino-cerebellar tracts. Furthermore, the fastigal nuclei and the vestibular cortex are believed to be involved in encoding internal postural model in space and self-motion. Some studies have even suggested projections of the fastigal nuclei to the motor cortex, parietal cortex, and multimodal visual areas (Takakusaki, 2017). According to this information, the cerebro-cerebellum might be involved in transforming a motor command into a prediction of the sensory outcome of a movement (Ishikawa et al., 2016), even during human locomotion.

In conclusion, we have researched auditory instructed locomotion trajectories with eyes open and closed with the aid of a virtual sound implementation. We observed head anticipation during the reproduction-task despite visual input, and visual imagery was reduced by using auditory instructions. This further supports the hypothesis that head anticipation has the function of motor anticipation. Moreover, our results also show that head locking to a target might occur regardless of the nature of the target itself. During the following-task, head anticipation might not have been observed due to constant search for the sound source. In the future, it would be relevant to study ocular behavior even when the eyes are closed. For this purpose, additional research on electro-oculography with the eyes closed is needed. Furthermore, it would be interesting to investigate head anticipation in a population with limited or complete absence of visual experience. Our paradigm in the eyes closed condition can be directly applied to such participants. This can provide deeper insight into the role of visual input in locomotion. Moreover, it would provide more evidence on whether head anticipation is innate or developed based on visual experience over time. Finally, a better understanding of the role of head anticipation in human gait control would be useful in several domains. These include motor rehabilitation after stroke, the development of prostheses, and other displacement mechanisms for people who lack this ability, and it will help to replicate bipedal walking in artificial agents.

## Data Availability

The raw data supporting the conclusions of this manuscript will be made available by the authors, without undue reservation, to any qualified researcher.

## Ethics Statement

The study was carried out at the university hospital of University of Tsukuba. All participants gave written informed consent in accordance with the Declaration of Helsinki. The protocol was approved by the ethical committee of the University of Tsukuba (2018R259).

## Author Contributions

FD and MP-H contributed the conception and together with HK and KS the design of the study. FD and HK prepared the custom software and collected the data. FD, MP-H, and HK performed the statistical analysis. FD and MP-H visualized the data. FD wrote the first draft of the manuscript. All authors contributed to manuscript revision, read, and approved the submitted version.

### Conflict of Interest Statement

The authors declare that the research was conducted in the absence of any commercial or financial relationships that could be construed as a potential conflict of interest.
